# A Cross-Layer, Anomaly-Based IDS for WSN and MANET

**DOI:** 10.3390/s18020651

**Published:** 2018-02-22

**Authors:** Amar Amouri, Salvatore D. Morgera, Mohamed A. Bencherif, Raju Manthena

**Affiliations:** 1 Department of Electrical Engineering, University of South Florida, Tampa, FL 33620, USA; aamouri@mail.usf.edu; 2 College of Computer & Information Sciences, King Saud University, P.O. Box 51178, Riyadh 11543, Saudi Arabia; mabencherif@ksu.edu.sa; 3 Jet Propulsion Laboratory, Pasadena, CA 91109, USA; raju.l.manthena@jpl.nasa.gov

**Keywords:** intrusion detection, MANET, WSN, decision trees, linear regression, accumulated measure of fluctuation (AMoF), finite sample size

## Abstract

Intrusion detection system (IDS) design for mobile adhoc networks (MANET) is a crucial component for maintaining the integrity of the network. The need for rapid deployment of IDS capability with minimal data availability for training and testing is an important requirement of such systems, especially for MANETs deployed in highly dynamic scenarios, such as battlefields. This work proposes a two-level detection scheme for detecting malicious nodes in MANETs. The first level deploys dedicated sniffers working in promiscuous mode. Each sniffer utilizes a decision-tree-based classifier that generates quantities which we refer to as correctly classified instances (CCIs) every reporting time. In the second level, the CCIs are sent to an algorithmically run supernode that calculates quantities, which we refer to as the accumulated measure of fluctuation (AMoF) of the received CCIs for each node under test (NUT). A key concept that is used in this work is that the variability of the smaller size population which represents the number of malicious nodes in the network is greater than the variance of the larger size population which represents the number of normal nodes in the network. A linear regression process is then performed in parallel with the calculation of the AMoF for fitting purposes and to set a proper threshold based on the slope of the fitted lines. As a result, the malicious nodes are efficiently and effectively separated from the normal nodes. The proposed scheme is tested for various node velocities and power levels and shows promising detection performance even at low-power levels. The results presented also apply to wireless sensor networks (WSN) and represent a novel IDS scheme for such networks.

## 1. Introduction 

The mobile adhoc network (MANET) is a type of wireless network that does not require infrastructure for its operation. This feature makes it very appealing for rapid deployment in harsh and challenging environments. There are many applications for these highly versatile networks. The most important applications of MANETs are in support of military special operations and civil emergencies. MANETs lack a centralized architecture which affects the level of security inside the network and increases vulnerability. Encryption helps to increase network security level but is not sufficient to protect against malicious intruders.

The rapid deployment of MANET nodes implies training data sets that are small vis-à-vis the problem dimensionality, which could lead to inaccuracies in the decision process [1]. The dynamic nature of such networks also leads to dataset drift [2]. These causes generally lead to errors made in the determination of malicious MANET nodes, but they also make the use of bilevel detection schemes of special interest. A bilevel detection scheme is meant to deploy a two-stage detection process, which uses dedicated sniffers to collect data and make decisions locally, while the global detection is performed via a supernode based on the data provided by the dedicated sniffers. 

**Figure 1:**
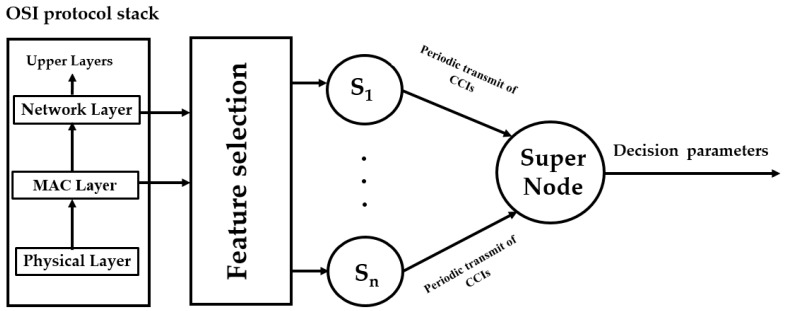
Simplified architecture of the proposed intrusion detection system (IDS).

The proposed intrusion detection scheme in this work is based on cross-layer feature collection and packet counts (from both the medium access control (MAC) and network layers) and is trained based on the network behavior under normal and malicious activity. To facilitate the understanding of the ideas presented in this paper, we preview the architecture used in the intrusion detection process. It is shown below in Figure 1, where dedicated sniffer nodes, $S_{i},\;i=1,\;\ldots ,\;n,$ capture information from several network layers and compute quantities which are referred to as correctly classified instances (CCI). The CCI used here is the ratio of the correctly classified instances obtained by the classifier to the total number of instances for both classes, normal and malicious. These quantities are then aggregated in a supernode which computes what we refer to as accumulated measure of fluctuation (AMoF).

A supervised approach to learning is adopted, having datasets labeled for both normal and malicious classes. The labeling was obtained based on the state of the network, that is, based on when there are no malicious nodes, and when some malicious nodes are deployed in the network. This approach will add robustness when compared to labeling based on node level. A node level labelling is prone to a change in the node functionality (different types of nodes). The classical detection measures such as true positive (TP) and true negative (TN), will have inaccurate inference regarding node status; instead, we used the accumulated variation of the CCIs calculated by the dedicated sniffers to generate a more solid inference regarding node status. A detailed discussion for dataset generation, labeling and detection algorithms, is presented in Section 4 and Section 5.

In this paper, the type of attack used to simulate the malicious activity is a black hole attack, which is network layer-based [3]. A more detailed discussion of the attack is found in Section 2. A non-specification-based IDS is adopted here, so features are collected and processed without taking special consideration of the specification of the attack behavior. We believe that this approach, given the nature of dataset preparation that is based on network behavior, rather than node behavior, and the non-specification-based detection, would lead to an IDS that exhibits robust behavior in the face of a variety of attacks. For example, in a black hole attack, the ratio of packets received to packets forwarded is an attack-specific feature that is not used here. In stage one, the detection process uses a decision tree (C4.5 or random forest) to produce CCIs, at the sniffer level, every reporting time (*Tr*). In stage two, the CCIs are sent to a fusion center (or supernode) that in turn applies an algorithm which calculates the AMoF for each sniffer’s data related to the node under test (NUT). This is done by calculating the accumulated variation of CCIs using a sliding window approach. An iterative slope fitting is performed in conjunction with the AMoF calculation process to establish a detection threshold. In the CCI calculations, to be described in the sequel, the usage of higher variance for larger groups of random variables has been favored to using smaller groups in the same sample space.

The C4.5 decision tree-based IDS is adopted due to its simplicity and reliability of performance [4,5,6,7,8,9,10]. An ensemble of decision trees, known as a random forest, is also adopted in this work due to its superior performance, especially when dealing with variability when compared to C4.5 decision trees. This point is very important, since we use the variation of the correctly classified instances (CCIs) as a major feature to differentiate between normal and malicious nodes. The random forest is considered to be one of the most effective data mining techniques [11]. A detailed comparison between different learning algorithms based on training time, computational complexity and resources consumption can be found in [12].

The data acquisition scheme used in the proposed IDS is based on promiscuous monitoring, and it relies on eavesdropping on the traffic activity of the neighboring nodes [13,14]. Dedicated sniffers use the collected data which consists of packet traces from MAC and network layers to build a model for normal and abnormal behavior at the node level.

This paper is divided into the following sections. Section 2 presents a brief introduction about the blackhole attack in MANET, while Section 3 presents mathematical modeling for the CCIs. It has been determined that, despite having a mathematical model for the CCIs, it is extremely difficult to find an analytical form for the AMoF. This motivates the utilization of a more tractable heuristic approach. The system architecture and a heuristic multilevel detection approach utilizing decision trees and linear regression is described in detail in Section 4. In Section 5, the experimental setup is explained in detail. Results and a discussion are provided in Section 6. A brief survey of related work is also presented in Section 7. Finally, Section 8 is devoted to conclusions and thoughts about future work.

## 2. AODV Routing and Blackhole Attack

The routing protocol used in this work is the adhoc on-demand distance vector (AODV), which is a reactive routing protocol that performs the discovery process between two nodes only when needed. There are three main control packets used in this protocol: route request (RREQ), route reply (RREP), and route error (RERR).

The black hole attack is a network layer-oriented attack that affects the routing pattern in the MANET by forging a fake RREP with a high-sequence number, thereby deceiving the network into believing that the attacking node has the shortest path to the destination [15]. Once the sender receives the forged RREP, the traffic behavior in the network changes by directing the traffic to a malicious node that drops the packets.

## 3. Mathematical Modeling of the CCIs

An analytical form of the AMoF is desired, in order to establish a proper detection threshold to distinguish between two classes, malicious and normal nodes. To obtain such a form, extensive work was conducted to obtain best distribution fitting of the accumulated CCIs, at the supernode, which represents the main block of the AMoF modeling. The data is collected in a matrix form, where the number of rows is equal to the number of reporting times *N*, and the number of columns is equal to the number of sniffing sensors *n*.

The matrix is populated with the CCIs obtained at every *Tr*, as shown in Table 1. The reporting time (*Tr*) is simply the simulation time divided by *N*, and is the time used to collect data by the sniffers to generate a CCI which is then sent to the supernode. An example of *Tr*/*Ts* calculation is shown in Section 5. The sample fitting was performed column-wise to show the best distribution fit for each sniffing sensor. The log-likelihood ratio is employed to decide the closest distribution to our data samples. The top three distributions that provided best fits were found to be the extreme value (EV), gamma, and Nakagami distributions. 

Algorithm 1 and the preceding paragraphs in Section 4, provide a detailed explanation and calculation of the AMoF, which are based on subtracting the consecutive CCIs for each sensor, taking the average CCI of the *n* sniffing sensors and finally, adding to the calculated sample from the previous iteration.

The extreme value distribution proved to be the best fitting distribution for a wide range of reporting time/sampling time (*Tr*/*Ts*) ratios based on the log-likelihood value. Samples of fitted distributions using a reporting time of *Tr* = 100 s and a sampling time of *Ts* = 10 s (for two extreme cases of node mobility and power level) are shown in Table 2 and Table 3. The data fitting for the first scenario with node velocity 1 m/s and power level 3 dBm is shown in Table 2. The other scenario is presented in Table 3, where the node velocity is 15 m/s and the power level is 7 dBm. Node 13, a normal node, and Node 19, a malicious node, are used to illustrate the best fit for the CCIs. In Table 2 and Table 3, the node under test (NUT) is indicated. The node speed and power levels are used to describe different scenarios in this work, for example, a node speed of 1 m/s and power level of 3 dBm is abbreviated as NS1P3. 

As mentioned earlier, an analytical model for the AMoF proved to be challenging; therefore, well-established heuristic methods were employed to develop a model and algorithm that calculates the AMoF. Given that the underlying distribution of the AMoF is extremely complex to obtain, we see it is important, at least, to model the main unit in calculating it, that is the CCI.

**Figure 2:**
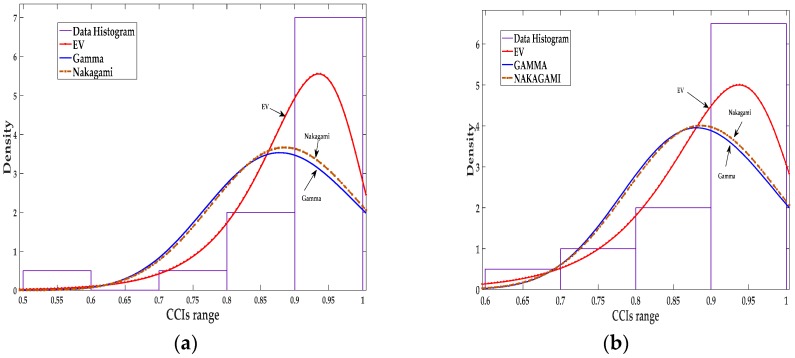
Data fitting for the CCIs of sensor 5: (**a**) Data fitting for three distributions, extreme value (EV), Gamma, and Nakagami in scenario NS1P3 for node under test (NUT) 13; (**b**) Data fitting for three distributions, EV, Gamma, and Nakagami in scenario NS1P3 for NUT 19.

The distribution fitting shown in Figure 2 shows that the EV distribution fits the CCIs better than the Gamma and Nakagami distributions. 

**Figure 3:**
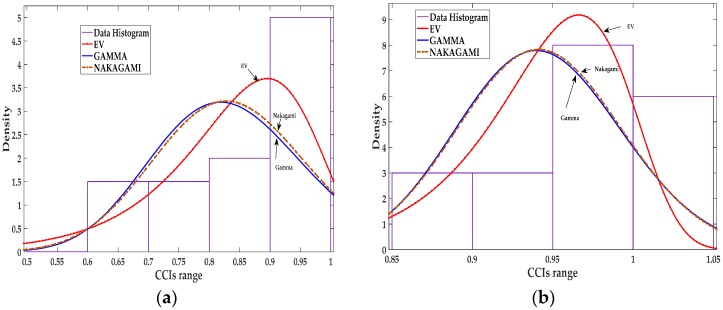
Data fitting for the CCIs of sensor 5: (**a**) Data fitting for three distributions, EV, Gamma, and Nakagami in scenario NS1P3 for NUT 13; (**b**) Data fitting for three distributions, EV, Gamma, and Nakagami in scenario NS15P7 for NUT 19.

The distribution fitting shown in Figure 3 shows that EV distribution fits the CCIs better than the gamma and Nakagami distributions. Gamma and Nakagami distribution fitting are very close (Figure 2 and Figure 3); this is expected considering the Nakagami distribution is a specific type of gamma distribution.

**Figure 4:**
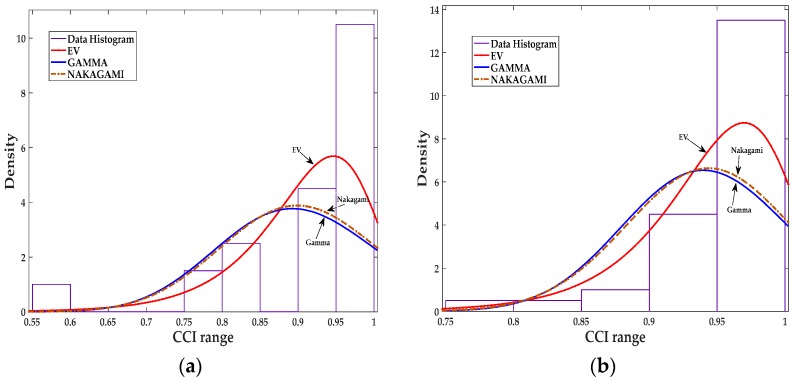
Data fitting for the CCIs of sensor 5: (**a**) Data fitting for three distributions, EV, Gamma, and Nakagami in scenario NS5P7 for NUT 13; (**b**) Data fitting for three distributions, EV, Gamma, and Nakagami in scenario NS5P7 for NUT 19.

The last set of distribution fitting results is presented in Table 4 and Figure 4, for scenario NS5P7, *T_r_* = 25 s, and *T_s_* = 5 s which has more fitting samples compared to the previous scenarios, in this case 2000/25 = 80 sample.

Again, the EV distribution shows to be the best fit compared with gamma and Nakagami distributions. All the results presented in this section are based on experimental setup described in Section 5.

## 4. System Architecture

**Figure 5:**
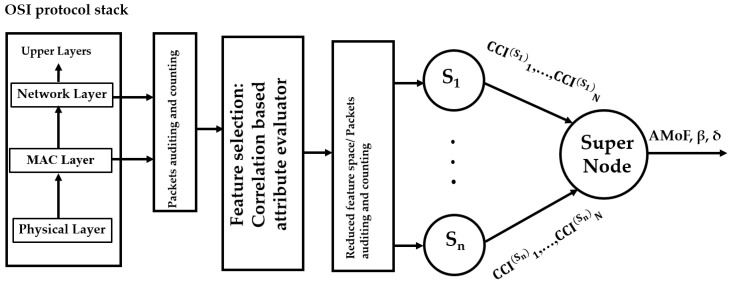
A two stage cross layer IDS.

The system shown in Figure 5 consists of *n* dedicated sniffers, $S_{i},\;i=1,\;\ldots ,\;n$. The packet traces are collected from the MAC and network layers related to the NUTs. The packets collected from the MAC layer are received and transmitted: request to send (RTS), clear to send (CTS), and acknowledgment (ACK). The packets collected from the network layers are received and transmitted: RREQ, RREP, and RERR. Both layers’ features are shown in Table 5. 

The variability in the consecutive CCIs is a key feature used to distinguish between normal and malicious nodes, where normal nodes have higher variability in consecutive CCIs. This idea is based on the smaller size population (which represents the number of malicious nodes in the network) vis-à-vis the larger-size population (which represents the number of normal nodes in the network).

The first stage of detection is conducted at the sniffer level, where each sniffer is a classifier and performs a local classification using a C4.5 decision tree or random forest. The outcome of this process is a CCI every *Tr* seconds, which is sent to the supernode for further processing. The simulation time is *N × Tr*, where the supernode performs the second stage of detection by applying an iterative slope (*β*) and threshold (*δ*) calculation based on linear regression for each NUT as shown in Algorithms 1 and 2. 

The AMoF is calculated in Algorithm 1 by applying a sliding window of size 1 on an *N × n* matrix, which is populated by the CCIs gathered about any NUT by the dedicated sniffers. This process if performed by subtracting the consecutive CCIs for each sensor, taking the average CCI of the *n* sniffing sensors, and finally adding the average of all the columns of the normalized absolute differences within each column, to the calculated sample from the previous iteration. The variable $Temp^{\left(S_{j}\right)}{}_{i}$ represents the subtraction of the consecutive CCIs, row-wise, where every column represents a number of sniffers. The absolute value is taken at each iteration and then divided by 100, which is the maximum possible difference between two consecutive CCIs. Finally, the $AMoF^{\left(S_{j}\right)}{}_{i}$ is calculated by finding the accumulated mean of the sniffers’ CCIs. The second algorithm uses linear regression to iteratively find the fitted slope and confidence intervals regarding the CCIs of each NUT. Note that is the number of instances received by the supernode, which is also the number of reporting times in the experiment.

Linear regression explains the dependency between the dependent variable *X* and independent variable *Y* as [16],

(1) $$Y_{i}=\beta_{0}+\beta_{1}X_{i}+\epsilon_{i},$$

where $\beta_{0}$ and $\beta_{1}$ are the model parameters. The errors $\epsilon_{i}$ are assumed to be independent $N\left(0,\sigma^{2}\right)$. The confidence interval for $\beta_{1}$ is given as

(2) $$b_{1}\pm \frac{t\left(n-2,1-\frac{\alpha }{2}\right)s}{{\left\{\sum^{}{\left(x_{i}-\overset{´}{x}\right)}^{2}\right\}}^{1/2}},$$

where $t\left(n-2,1-\frac{\alpha }{2}\right)$ is the $100\left(1-\frac{\alpha }{2}\right)$ percentage point of a t-distribution with (n−2) degrees of freedom and the residual sum of squares $s^{2}$. The confidence of the slope in (2) is *C* as in Algorithm 2. Once enough AMoF samples have been generated (three samples suffice), the iterative regression calculates the fitted slope $\beta_{1}$, including its upper and lower bounds. The threshold is calculated first by finding an initial threshold $\left(\delta^{*}\right)$ which is the difference between the maximum of the upper bounds and the minimum of the upper bounds of all NUTs divided by two and added to the minimum of the upper bounds for that iteration. 

**Algorithm 1** Calculating the AMoF for malicious and normal nodes1:**Input:** $CCI^{\left(S_{n}\right)}{}_{1}$*,………,*
$CCI^{\left(S_{n}\right)}{}_{N}$*,*2:**Output:** AMoF3:**At the super node**4: ∀node∈NUT5:Receive $CCI^{\left(S_{n}\right)}{}_{1}$,………,$\;CCI^{\left(S_{n}\right)}{}_{N}$ S.T $CCI^{\left(NUT\right)}\;is\;N\times n$6:Initialize $Temp^{\left(S_{j}\right)}$, Norm_$Temp^{\left(S_{j}\right)}{}_{i}$, $Sigma^{\left(S_{j}\right)}{}_{i},\;AMoF^{\left(S_{j}\right)}{}_{i}$7:**for**
*i* = 1 *to*
N
**do**8:    **for**
*j* = 1 *to* n **do**9:   $Temp^{\left(S_{j}\right)}{}_{i}$←$|(CCI^{\left(S_{j}\right)}{}_{i+1}-CCI^{\left(S_{j}\right)}{}_{i})|+Temp^{\left(S_{j}\right)}{}_{i-1}$10$Norm\_Temp^{\left(S_{j}\right)}{}_{i}$**←**$Temp^{\left(S_{j}\right)}{}_{i}$/10011:    **end for**12:  $\;AMoF^{\left(S_{j}\right)}{}_{i}$**←**
$(Norm\_Temp^{\left(S_{j}\right)}{}_{i}/n$) +$\;\;AMoF^{\left(S_{j}\right)}{}_{i-1}$13:**end for**

The permanent threshold (δ) value is adopted once a small number (say 3) of consecutive initial thresholds achieve the criterion |∆|. 

**Algorithm 2** Calculating the fitted slope (β) for every NUT, setting the detection threshold (δ)1:**Input:** $AMoF^{\left(NUT\right)}{}_{1}$*,………,*
$AMoF^{\left(NUT\right)}{}_{N-1}$2:**Output:** fitted slope (β), detection threshold (δ)3:**At the super node:**4: ∀node∈NUT where the number of elements in **NUT** = *l*5:Receive $AMoF^{\left(NUT\right)}{}_{1}$*,..,*
$AMoF^{\left(NUT\right)}{}_{N-1}$ S.T $AMoF^{\left(NUT\right)}$ is l×(N−1)6:**for** k = 1 to N−1
**do**7:    **for**
*j* = 1 *to NUT*
**do**8:    **If** k ≥ 3 ** then**9:     Find $\beta_{k}$ by solving (1)10:    Find $C_{k}$ by solving (2)11:    Find initial threshold $\delta_{k}^{*}$ = $\left(\frac{max\left(C_{k}\right)-min\left(C_{k}\right)}{2}\right)+min\left(C_{k}\right)$12:     **If**
$\delta_{k}^{*}-\delta_{k-1}^{*}\leq \left|∆\right|$13:       $\delta_{k}\leftarrow \delta_{k}^{*}$14:        **If**
$\delta_{k}>\delta$15:         node is normal16:        **else**17:         node is malicious18:   **end for**19:**end for**

The normal nodes are those which have a higher upper bound value than the permanent threshold. In other words, normal nodes maintain higher fluctuation in the received CCIs compared to the malicious nodes. 

## 5. Experimental Setup

In the network of 30 nodes, there exist five data sources transmitting data to five destination nodes (sinks). All other nodes act as routers following an AODV protocol. In the experiment, UDP (user datagram protocol) connection was used and data traffic of constant bit rate (CBR) was applied between source node to destination nodes. The network used in this experiment has a data rate of 2Mb/s (802.11b) and assumes a Friis loss model [17]. Different power levels and node velocities are studied in the paper as shown in Table 4.

The raw data were collected by simulating a network of thirty mobile nodes using the widely adopted discrete-event network simulator NS-3. A node’s mobility follows the random way point (RWP) mobility model. Five of the nodes are designated as dedicated sniffers that collect data from the neighboring nodes promiscuously. They then generate CCIs, which in turn are sent to the supernode that performs the AMoF algorithm that includes a linear regression of the MoF so that it can detect the malicious nodes. The first level detection is at the sniffers’ level, where each sniffer generates a CCI at each Tr. Simple, yet effective, machine-learning algorithms have been adopted; most notably, the C4.5 decision tree or random forest. A supervised learning approach is adopted, which means simulations for both normal and malicious dataset retrieval are needed.

The number of sniffers was chosen as a tradeoff between the following considerations:

1. The Area covered is 1000 m by 1000 m, which requires a sufficient number of sniffers, especially when having low connectivity scenarios, e.g., node speed = 15 m/s, and power level 3 dBm (NS15P3).
2. The variability of data collected from a small number of sniffers will on average be higher than collected by a larger number of sniffers.
3. The ability to provide effective intrusion detection for a small number of blackhole malicious nodes, i.e., the expectation is: the larger the number of black holes, the larger the required number of sniffers.

For those reasons, the number of sniffers chosen was five, to provide effective area coverage, moderate variability, and effective operation for up to three black holes.

The first set of simulations was performed to obtain the behavior of the network under normal conditions (no black holes deployed), by running the experiment for 2000 s each time for twenty different seeds, to have a better statistical average, for every single scenario. In the second set of simulations, a malicious condition was obtained by deploying three black holes in the network and running the simulations as in the normal case for another twenty seeds for each single scenario. The power levels, the node’s mobility, and other simulation parameters are listed in Table 6.

The extreme case scenarios adopted in this paper are chosen such that they span a diversity of node velocities and power levels. These scenarios are: NS1P3, NS1P7, NS15P3, and NS15P7. The abbreviation NS1P3, for example, means node velocity 1m/s and power level of 3dBm. The model of normal and malicious states is obtained at every reporting time and is used to train the classifiers which, in turn, generate the CCIs. Different reporting times are used: 50 s, 100 s, and 200 s, and within each reporting time, different sampling times are used such as 5 s, 10 s, 20 s, and 40 s, depending on the length of the reporting period. For example, if we have a reporting time of 50 s, the sampling time used is 5 s, or 10 s. the ratio between *Tr* and *Ts* is chosen to have at least five instances in each class (normal or malicious). A reporting time of 200 s and sampling time of 5 s, will give 40 instances for each class. 

A static set of features collected from the MAC and network layers are used for the detection process using a C4.5 decision tree or random forest, as shown in Table 5. To avoid feature redundancy and for computational efficiency, a subset of the features is selected using a correlation-based feature selection technique called a correlation attribute evaluator, where the features are ranked according to their relative weights; the higher the weight, the more influential is the feature on the decision process. The top six features were chosen in every scenario for every NUT. The classifiers are validated using a ten-fold cross validation process. This process divides the labeled data into ten equal folds and uses nine folds for training and one-fold for testing. The process continues in a circular way, and resulting parameters are averaged over ten. 

At each reporting time (*Tr*), the sniffers generate a set of TN, TP, FP, FN, which constitutes the confusion matrix. The CCI which is the sum of the TP and TN, showed via the calculation of its variation in timely manner, to be a better measure for differentiating between malicious and normal nodes, this is mainly due to the labeling of the data set that is based on network behavior, by having all normal nodes for the normal labeling process, and three malicious nodes when obtaining the malicious labeling. This approach was meant to give higher robustness when having networks with different nodes functionalities or roles.

A sample of performance measures were added in Appendix A. Those are related to the scenarios in Section 6. The results are related to one dedicated sniffer regarding two nodes, node 13 (normal) and node 21 (malicious). Three instances for each scenario are taken from the beginning, middle, and the end of the simulation.

## 6. Results and Discussion

**Figure 6:**
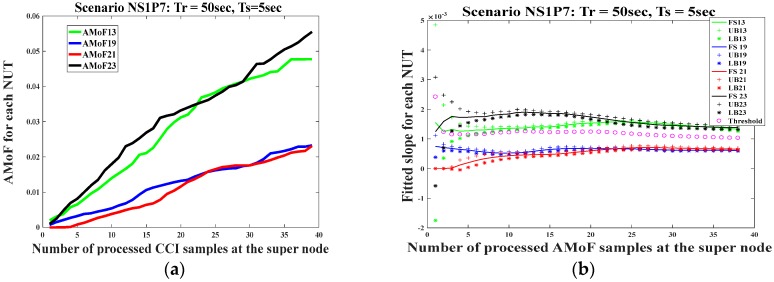
The AMoF for different nodes and the fitted slope for those nodes for scenario NS1P7 (*Tr* = 50 s, *Ts* = 5 s): (**a**) The AMoF for different TNs; (**b**) The fitted slope, and its confidence for different NUTs.

**Figure 7:**
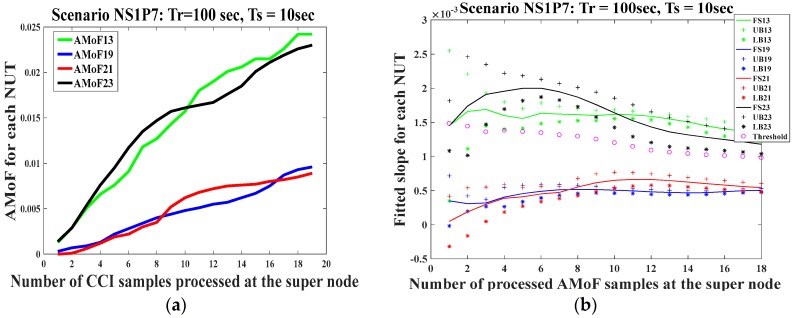
The AMoF for different nodes and the fitted slope for those nodes for scenario NS1P7 (*Tr* = 100 s, *Ts* = 10 s): (**a**) The AMoF for different NUTs; (**b**) The fitted slope, and its confidence for different NUTs.

Extensive simulations for different scenarios having different power levels and node velocities were conducted. The scenarios presented here are representative of the performance for the maximum and minimum cases both in power and velocity. The results of the AMoF and detection threshold for scenario NS1P7 with *Tr* = 50 s and *Ts* = 5 s are shown in Figure 6. The results of the AMoF and detection threshold for scenario NS1P7 with *Tr* = 100 s and *Ts* = 10 s are shown in Figure 7. Both cases exhibit good detection behavior in which the threshold permits discrimination between normal and malicious nodes starting from the sixth iteration which corresponds to 300 s of simulation time for the first case. The detection threshold criterion, which represents the difference between consecutive fitted slopes, is chosen after a certain number of iterations that achieve small fluctuation in the consecutive fitted slopes, showing stable behavior; |∆| = 10^−3^ was picked for this purpose. 

Similarly, in the second case, the detection threshold gives good results at the fifth iteration, which corresponds to 500 s of simulation. The abbreviations in part b in each set of Figures refer to the fitted slope (FS), upper bound (UB), and lower bound (LB) intervals for each NUT.

**Figure 8:**
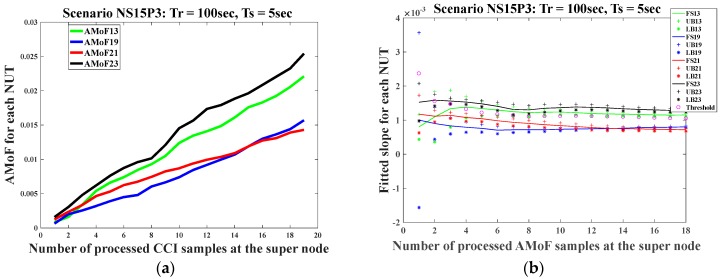
The AMoF for different nodes and the fitted slope for those nodes for scenario NS15P3 (*Tr* = 100 s, *Ts* = 5 s): (**a**) The AMoF for different NUTs; (**b**) The fitted slope, and its confidence for different NUTs.

The last scenario shown is NS15P3 with *Tr* = 100 s and *Ts* = 5 s in Figure 8. The performance in this case shows a major decline, as the detection threshold lies very close to the margins of normal and malicious nodes, making the detection process very difficult. The ability of the dedicated sniffers to capture firsthand packets related to the TNs is highly affected by having those nodes within their sense range. This is reflected on building detection models that generates low variability in the CCIs leading to tight detection margins as shown in Figure 8b. According to [18], the nodes with low velocity and high power would have higher connectivity. Analytical simulation results for the probability of detection vs. sensing range of nodes in a wireless sensor network was presented in [19] and shows a higher detection probability as the sensing range increases. This will impact the amount of data collected by the dedicated sniffers; the higher the connectivity, the more packets collected. This helps to build more accurate models that will then allow the generation of more accurate decisions.

It is important to mention that the detection performance for the whole IDS is determined at the supernode level. The detection rate reaches 100% once the detection criterion is achieved. This would require more time to reach in some challenging scenarios, as shown in Figure 8 where the connectivity of the nodes is at its lowest.

## 7. Related Work

In this section, we present a brief review about the use of decision trees, random forest, and linear regression in IDS design. The lack of a standard dataset in the field of IDS design for MANETs makes an accurate comparison process between different systems difficult. This situation is not present in legacy networks, where datasets such as the KDD99 have been used extensively. One of the earliest efforts in the field of IDS design in MANET using C4.5 decision trees is the work of Huang and Lee [20]. They used a set of statistical features which can be derived from routing tables and then applied C4.5 to detect normal versus abnormal behavior. Huang et al. [21] proposed a “cross-feature analysis” method to capture the inter-feature correlation patterns in normal traffic. An intrusion detection and adaptive response mechanism (IDAR) was proposed by Nadeem and Howarth [22]. All nodes in the network operate as one of the three roles of manager node (MN), cluster heads (CH), and cluster nodes (CNs). A decision tree in the MN with the calculated confidence on attack detected (COA) and network performance degradation (NPD) values are used to identify the intrusion response action. A distributed combined authentication and intrusion detection with data fusion in MANET was proposed by Bu et al. [23], who used a decision tree to organize the attack signatures into a tree structure. Silva et al. [24] used Linear Regression and Variance Analysis (LRVA), a multi-step-ahead prediction where the fake parameters broadcasted in the network were detected, with the malicious/misbehaving nodes identified.

## 8. Conclusions and Future Work

A new IDS design based on a two-stage detection process is proposed in this paper. The use of the AMoF as a novel feature showed promising results especially for small size datasets; this is inherent to MANET operation, particularly in demanding environments where rapid decisions must be made. The inaccuracies of detection in stage one are compensated for in stage two, by taking advantage of the variability of the CCIs for normal and malicious nodes. The idea is based on the variance of two distinct sizes of random variable populations, i.e., the normal and malicious node populations, where the variance is higher for the larger normal node population (in our case, 90% of the total nodes). This scheme, as all others we are aware of, will face higher challenges when exposed to comparably sized populations. The results show that for low-node velocity and high power transmitted, e.g., node velocity of 1 m/s and power level of 7dBm (NS1P7), different ratios of reporting time to sampling time, *Tr*/*Ts*, reveal well-separated regions that facilitate the detection process. This is related to a high-node connectivity. The scenario of high-node velocity and low-transmitted power, e.g., NS15P3, shows less detection capability. This is related to low node connectivity, a regime in which MANET performance always degrades. Our solution is rather robust across a range of node speed and transmit power regimes, and the goal of future work is to further improve on this.

Since much of the underlying probability distribution is a priori incompletely known, the classifier must continually adapt when pattern class structures change [25]. Therefore, MANET systems require continuous training to obtain reasonable detection results. However, the ratio between sample size to feature size in this case is close to three, which according to [26] demonstrates that the design-set error rate is an extremely biased estimate of either the Bayes or test-set error rate. Computationally demanding techniques can be used to reduce the effect of finite sample size, such as Monte-Carlo resampling, cross validation, etc., which may have problems when dealing with correlated features resulting in inaccurate decisions.

Our approach deals with the stringent requirements facing WSN and MANET deployment, such as the limited size of training datasets and computational power availability. A new scheme based on the CCI, which includes information on both the true positive (TP) and the true negative (TN), is used due to the fact of having very limited training instances. Such a finite sample size generally limits the accuracy of classifiers as compared to situations where training instances are much larger, for example, in legacy or fixed networks. It is the variation of the CCI values that is of main interest, since the values of the CCIs, including its corresponding TP and TN at any single reporting time (*Tr*), generates inaccurate decisions.

In addition, a new scheme will be examined in future work that is based on incremental data acquisition for decision-making to alleviate the need of the second stage. Specifically, a more adaptive feature selection scheme will be tested, which fits the dynamic nature of the MANET. Testing the proposed IDS on different type(s) of malicious activity, such as denial of service, is needed to verify its robustness under wider range of attacks. Moreover, a wider range of classifiers will be utilized as well. These changes are expected to improve the decision accuracy associated with the nodes under test. The results presented here also apply to wireless sensor networks (WSN) and represent a novel intrusion detection scheme for such networks.

## Figures and Tables

**Table 1:** A sample table for the collected correctly classified instances (CCIs) at the super node every *Tr.*

	Sniffers	
Correctly Classified Instances (CCIs) for every repotting time *T_r_*	$S_{1}$	$S_{n}$
	$CCI_{1}^{\left(S_{1}\right)}$	$CCI_{1}^{\left(S_{n}\right)}$
	$CCI_{N}^{\left(S_{1}\right)}$	$CCI_{N}^{\left(S_{n}\right)}$

**Table 2:** The log-likelihood for three different density fits in scenario NS1P3.

NS1P3, *Tr* = 100 s, *Ts* = 10 s			
NUT: 13		NUT: 19	
Log-likelihood for sensor 5		Log-likelihood for sensor 5	
EV	21.4374	EV	20.0272
Gamma	15.2119	Gamma	17.4694
Nakagami	16.0349	Nakagami	18.1008

**Table 3:** The log-likelihood for three different density fitting in scenario NS15P7.

NS15P7, *Tr* = 100 s, *Ts* = 10 s.			
NUT: 13		NUT: 19	
Log-likelihood for sensor 5		Log-likelihood for sensor 5	
EV	14.4636	EV	32.4864
Gamma	13.1913	Gamma	31.0568
Nakagami	13.4487	Nakagami	31.2517

**Table 4:** The log-likelihood for three different density fitting in scenario NS5P7.

NS5P7, *T_r_* = 25 s, *T_s_* = 5 s			
NUT: 13		NUT: 19	
Log-likelihood for sensor 5		Log-likelihood for sensor 5	
EV	43.8455	EV	61.8714
Gamma	33.0146	Gamma	55.1129
Nakagami	34.326	Nakagami	56.2025

**Table 5:** Cross Layer Features.

**Mac Layer**	Tx/Rx	Tx/Rx	Tx/Rx	
	RTS	CTS	ACK	
**Network Layer**	Tx/Rx	Tx/Rx	Tx/Rx	Tx/Rx
	RREQ	RREP	RERR	Hello messages

**Table 6:** Simulation Parameters.

**No. of Nodes**	30
**Field area**	1000 m × 1000 m
**Node speed**	1 m/s, 5 m/s, 10 m/s 15 m/s
**Simulation time**	2000 s
**Power levels**	3 dBm, 7 dBm
**MAC layer protocol**	802.11 b
**Routing protocol**	AODV
**Mobility model**	RWP
**Reporting time (Tr)**	50 s, 100 s, 200 s
**Sampling time (Ts)**	5 s, 10 s, 20 s, 40 s
**Nodes Under Test NUTs**	13, 19, 21, and 23
**Traffic source**	CBR (UDP)

## Appendix A

In this appendix, a sample of results for the random forest classifier corresponding to the scenarios in Section 6 are presented. The results are related to one dedicated sniffer regarding two nodes, node 13 (Normal) and node 21 (malicious). Three instances for each scenario are taken from the beginning, middle, and the end of the simulation.

The results in Table A1a, NUT 13, *Tr* = 50 s, *Ts* = 5 s at the beginning of the simulation period in scenario NS1P7. The CCI value is 65%.

The results in Table A1b, NUT 21, *Tr* = 50 s, *Ts* = 5 s at the beginning of the simulation period in scenario NS1P7. The CCI value is 100%.

**Table A1:** (**a**) Detailed Accuracy by Class for NUT 13; (**b**) Detailed Accuracy by Class for NUT 21.

(**a**)								
**TPR**	**FPR**	**Precision**	**Recall**	**F-Measure**	**MCC**	**ROC Area**	**PRC Area**	**Class**
0.7	0.4	0.636	0.7	0.667	0.302	0.725	0.726	Normal
0.6	0.3	0.667	0.6	0.632	0.302	0.725	0.794	Malicious
(**b**)								
**TPR**	**FPR**	**Precision**	**Recall**	**F-Measure**	**MCC**	**ROC Area**	**PRC Area**	**Class**
1	0	1	1	1	1	1	1	Normal
1	0	1	1	1	1	1	1	Malicious

The results in Table A2a corresponds to NUT 13, *Tr* = 50, *Ts* = 5 s at the middle simulation period in scenario NS1P7. The CCI value is 90%.

The results in Table A2b corresponds to NUT 21, *Tr* = 50 s, *Ts* = 5 s at the middle simulation period in scenario NS1P7. The CCI value is 100%.

**Table A2:** (**a**) Detailed Accuracy by Class, for NUT 13; (**b)** Detailed Accuracy by Class for NUT 21.

(**a**)								
**TPR**	**FPR**	**Precision**	**Recall**	**F-Measure**	**MCC**	**ROC Area**	**PRC Area**	**Class**
0.9	0.1	0.9	0.9	0.9	0.8	0.945	0.927	Normal
0.9	0.1	0.9	0.9	0.9	0.8	0.945	0.963	Malicious
(**b**)								
**TPR**	**FPR**	**Precision**	**Recall**	**F-Measure**	**MCC**	**ROC Area**	**PRC Area**	**Class**
1	0	1	1	1	1	1	1	Normal
1	0	1	1	1	1	1	1	Malicious

The results in Table A3a corresponds to NUT 13 *Tr* = 50 s, *Ts* = 5 s at the end of the simulation period in scenario NS1P7. The CCI value is 100%.

The results in Table A3b corresponds to NUT 21, *Tr* = 50 s, *Ts* = 5 s at the end of the simulation period in scenario NS1P7. The CCI value is 75%.

**Table A3:** (**a**) Detailed Accuracy by Class, for NUT 13; (**b**) Detailed Accuracy by Class, for NUT 21.

(**a**)								
**TPR**	**FPR**	**Precision**	**Recall**	**F-Measure**	**MCC**	**ROC Area**	**PRC Area**	**Class**
1	0	1	1	1	1	1	1	Normal
1	0	1	1	1	1	1	1	Malicious
(**b**)								
**TPR**	**FPR**	**Precision**	**Recall**	**F-Measure**	**MCC**	**ROC Area**	**PRC Area**	**Class**
0.8	0.3	0.727	0.8	0.762	0.503	0.72	0.672	Normal
0.7	0.2	0.778	0.7	0.737	0.503	0.72	0.766	Malicious

The results in Table A4a corresponds to NUT 13, *Tr* = 100 s, *Ts* = 10 s at the beginning of the simulation period in scenario NS1P7. The CCI value is 75%.

The results in Table A4b corresponds to NUT 21, *Tr* = 100 s, *Ts* = 10 s at the beginning of the simulation period in scenario NS1P7. The CCI value is 100%.

**Table A4:** (**a**) Detailed Accuracy by Class, for NUT 13; (**b**) Detailed Accuracy by Class, for NUT 21.

(**a**)								
**TPR**	**FPR**	**Precision**	**Recall**	**F-Measure**	**MCC**	**ROC Area**	**PRC Area**	**Class**
0.8	0.3	0.727	0.8	0.762	0.503	0.72	0.672	Normal
0.7	0.2	0.778	0.7	0.737	0.503	0.72	0.766	Malicious
(**b**)								
**TPR**	**FPR**	**Precision**	**Recall**	**F-Measure**	**MCC**	**ROC Area**	**PRC Area**	**Class**
1	0	1	1	1	1	1	1	Normal
1	0	1	1	1	1	1	1	Malicious

The results in Table A5a corresponds to NUT 13, *Tr* = 100, *Ts* = 10 s at the middle of the simulation period in scenario NS1P7. The CCI value is 65%.

The results in Table A5b corresponds to NUT 21, *Tr* = 100 s, *Ts* = 10 s at the middle of the simulation period in scenario NS1P7. The CCI value is 80%.

**Table A5:** (**a**) Detailed Accuracy by Class, for NUT 13; (**b**) Detailed Accuracy by Class for NUT 21.

(**a**)								
**TPR**	**FPR**	**Precision**	**Recall**	**F-Measure**	**MCC**	**ROC Area**	**PRC Area**	**Class**
0.7	0.4	0.636	0.7	0.667	0.302	0.785	0.837	Normal
0.6	0.3	0.667	0.6	0.632	0.302	0.785	0.778	Malicious
(**b**)								
**TPR**	**FPR**	**Precision**	**Recall**	**F-Measure**	**MCC**	**ROC Area**	**PRC Area**	**Class**
0.7	0.1	0.875	0.7	0.778	0.612	0.97	0.97	Normal
0.9	0.3	0.75	0.9	0.818	0.612	0.97	0.977	Malicious

The results in Table A6a corresponds to NUT 13 *Tr* = 100 s, *Ts* = 10 s at the end of the simulation period in scenario NS1P7. The CCI value is 100%.

The results in Table A6b corresponds to NUT 21, *Tr* = 100 s, *Ts* = 10 s at the end of the simulation period in scenario NS1P7. The CCI value is 90%.

**Table A6:** (**a**) Detailed Accuracy by Class, for NUT 13; (**b**) Detailed Accuracy by Class, for NUT 21.

(**a**)								
**TPR**	**FPR**	**Precision**	**Recall**	**F-Measure**	**MCC**	**ROC Area**	**PRC Area**	**Class**
1	0	1	1	1	1	1	1	Normal
1	0	1	1	1	1	1	1	Malicious
(**b**)								
**TPR**	**FPR**	**Precision**	**Recall**	**F-Measure**	**MCC**	**ROC Area**	**PRC Area**	**Class**
0.8	0	1	0.8	0.889	0.816	0.885	0.932	Normal
1	0.2	0.833	1	0.909	0.816	0.885	0.786	Malicious

The results in Table A7a corresponds to NUT 13, *Tr* = 100 s, *Ts* = 5 s at the beginning of the simulation period in scenario NS15P3. The CCI value is 67.5%.

The results in Table A7b corresponds to NUT 21, *Tr* = 100 s, *Ts* = 5 s at the beginning of the simulation period in scenario NS15P3. The CCI value is 95%.

**Table A7:** (**a**) Detailed Accuracy by Class, for NUT 13; (**b**) Detailed Accuracy by Class, for NUT 21.

(**a**)								
**TPR**	**FPR**	**Precision**	**Recall**	**F-Measure**	**MCC**	**ROC Area**	**PRC Area**	**Class**
0.7	0.35	0.667	0.7	0.683	0.35	0.77	0.789	Normal
0.65	0.3	0.684	0.65	0.667	0.35	0.77	0.778	Malicious
(**b**)								
**TPR**	**FPR**	**Precision**	**Recall**	**F-Measure**	**MCC**	**ROC Area**	**PRC Area**	**Class**
1	0.1	0.909	1	0.952	0.905	0.99	0.99	Normal
0.9	0	1	0.9	0.947	0.905	0.99	0.992	Malicious

The results in Table A8a corresponds to NUT 13, *Tr* = 100 s, *Ts* = 5 s at the middle of the simulation period in scenario NS15P3. The CCI value is 77.5%.

The results in Table A8b corresponds to NUT 21, *Tr* = 100 s, *Ts* = 5 s at the middle of the simulation period in scenario NS15P3. The CCI value is 100%.

**Table A8:** (**a**) Detailed Accuracy by Class for NUT 13; (**b**) Detailed Accuracy by Class, for NUT 13.

(**a**)								
**TPR**	**FPR**	**Precision**	**Recall**	**F-Measure**	**MCC**	**ROC Area**	**PRC Area**	**Class**
0.7	0.15	0.824	0.7	0.757	0.556	0.785	0.85	Normal
0.85	0.3	0.739	0.85	0.791	0.556	0.785	0.705	Malicious
(**b**)								
**TPR**	**FPR**	**Precision**	**Recall**	**F-Measure**	**MCC**	**ROC Area**	**PRC Area**	**Class**
1	0	1	1	1	1	1	1	Normal
1	0	1	1	1	1	1	1	Malicious

The results in Table A9a corresponds to NUT 13 *Tr* = 100 s, *Ts* = 5 s at the end of the simulation period in scenario NS1P7. The CCI value is 77.5%.

The results in Table A9b corresponds to NUT 13 *Tr* = 100 s, *Ts* = 5 s at the end of the simulation period in scenario NS1P7. The CCI value is 90%.

**Table A9:** (**a**) Detailed Accuracy by Class, for NUT 13; (**b**) Detailed Accuracy by Class, for NUT 13.

(**a**)								
**TPR**	**FPR**	**Precision**	**Recall**	**F-Measure**	**MCC**	**ROC Area**	**PRC Area**	**Class**
0.75	0.2	0.789	0.75	0.769	0.551	0.794	0.79	Normal
0.8	0.25	0.762	0.8	0.78	0.551	0.794	0.749	Malicious
(**b**)								
**TPR**	**FPR**	**Precision**	**Recall**	**F-Measure**	**MCC**	**ROC Area**	**PRC Area**	**Class**
0.9	0.1	0.9	0.9	0.9	0.8	0.97	0.973	Normal
0.9	0.1	0.9	0.9	0.9	0.8	0.97	0.971	Malicious
